# Remarks on the Asserted Muscularity of the Arteries

**Published:** 1825-02

**Authors:** William Mackenzie

**Affiliations:** Andersonian Professor of Anatomy and Surgery, and one of the Surgeons to the Glasgow Eye Infirmary.


THE LONDON

### Medical and Physical Journal.

No. 2 of Vol. LIII.] FEBRUARY, 1825. [No. 312.

For many fortunate discoveries in medicine, and for the detection of numerous errors, the world is indebted to the rapid circulation of Monthly Journals; and there never existed any work, to which the Faculty, in Europe and America, were under deeper obligations, than to the Medical and Physical Journal of London, now forming a long, but an invaluable, series.—RUSH.

### ORIGINAL COMMUNICATIONS,

### SELECT OBSERVATIONS, &c.

ART. I.—*Remarks on the asserted Muscularity of the Arteries.* By WILLIAM MACKENZIE, Andersonian Professor of Anatomy and Surgery, and one of the Surgeons to the Glasgow Eye Infirmary.

IN the third article of the Edinburgh Medical and Surgical Journal for October, 1824, this subject has been brought forward, with not a few inaccuracies: these it is the object of the following remarks to point out; for, on such a subject as the powers which circulate the blood, or indeed on any subject, it is plainly impossible to reason justly, unless the premises be true. In no science more than in physiology, ought the truth, the whole truth, and nothing but the truth, to be our object; for on our physiology depend many of our practical opinions in medicine and surgery, and, consequently, in many cases, the lives of those who entrust themselves to our care.

In the first sentence of his paper, the writer in the Journal above mentioned entirely confounds contractility and muscularity; and, throughout all his reasonings, he wishes us to believe that, if the arteries are contractile, they must be muscular. But there is a difference, acknowledged by all physiologists, between contractility of tissue and muscular contraction; and, while the former of these properties is universally allowed to belong to the arterial tunics, the latter is by many denied to them. Every one knows that the veins contract,—that the cellular substance and the skin contract. Are we to conclude that the contractions of these parts are performed by muscular fibres? Contractility of tissue is so plainly distinguishable from muscular contractility, and so generally distinguished by physiologists, that to confound them appears not a little extraordinary.

In order to avoid any ambiguity which might arise from using technical terms in a vague sense, I shall here shortly illustrate the meaning of the words, by which, in the following remarks, I shall express the real and asserted properties of arteries.

1. *Elasticity.*—When I press together the sides of a portion of the aorta, taken from the dead body, on merely withdrawing the force with which I had compressed it, it opens again, and returns to its former circular state, by means of its elasticity.

2. *Extensibility of tissue.*—When I introduce into the mouth of a divided artery the points of a pair of forceps, and allow the blades of the instrument to separate, I dilate the artery, and show its extensibility of tissue. The same property is shown when, with the tenaculum, I drag forth an artery on the face of a stump.

3. *Contractility of tissue.*—When I inclose a portion of an artery, in the living subject, between two ligatures, and puncture the artery between, the vessel immediately empties itself of the greater part of its contents, by means of its contractility of tissue. By the same property, the artery on the face of a stump retires among the muscles, when the tenaculum is withdrawn.

4. *Extensibility of growth.*—The arteries of the uterus during pregnancy are greatly enlarged, not by any mere mechanical dilatation and elongation, but by a vital process, performed by means of their nutrient capillaries. This extensibility of growth we see illustrated also in the formation of tumors, the healing of wounds, and in various other instances.

5. *Contractility of growth.*—When the uterine arteries shrink after parturition,—when the ductus arteriosus shrinks to a ligament,—or an artery shrinks after the operation for aneurism, an opposite vital process is performed by the absorbents of the vessels, illustrating what we may call contractility of growth.

Tubes not possessing elasticity and the two varieties of extensibility and contractility above mentioned, could not have answered the purposes of arteries. They could not have accommodated themselves, either to the changes of the body in locomotion, or to the quantity of blood they might at different times contain, or to the variable demand for blood in different parts of the body.

It is disputed whether arteries possess these two properties:—

1. *Muscular irritability.*—On taking up a limb after it is amputated, if we prick the muscles with the point of a scalpel, they are seen to quiver,—or, in other words, they betray muscular irritability.

2. *Muscular contractility.*—When the heart is laid bare in a living animal, we observe the auricles and ventricles alternately dilating and contracting. The latter is acknowledged by all to be the effect of the muscular contractility of the fleshy fibres of the heart.

It is further necessary, in order that we may know of what we are speaking, to distinguish the *arteries* from the *capillaries.*

The arteries are the counterpart of the veins; the capillaries are the counterpart of the absorbents. The arteries nourish nothing; the capillaries nourish every part. The arteries are considerable in size, and wind merely in the interstices of our organs; the capillaries are as small as hairs, and enter into the essential substance of every part. The arteries uniformly carry red blood; the blood of the capillaries is in many parts colorless.

*Arguments against the Muscularity of Arteries.*

I. The first argument against the muscularity of the arteries, which the writer in the Journal alluded to professes to examine, is " that muscular fibres have never been detected in arteries."

We might be led to suppose that, under this head, he would, first of all, favour us with a test, or criterion, by which muscular fibre might be discriminated from other tissues, and then apply this test to the substance in dispute. Any thing of this sort, however, he has declined to attempt.

There may be no single test by which dead muscular fibre can be absolutely distinguished from the other fibres which compose the body. The following, however, are certain physical differences, existing between the middle coat of the human arteries, and what are by all acknowledged to be muscular fibres in other parts of the human body.

1. Muscular fibres are universally round, soft, lax, almost quite inelastic, very extensible, and with three-fourths of their weight formed of fluid. The arterial fibres are, on the contrary, flat, dry, very elastic, fragile, and easily snap through on traction; in all these respects differing widely both from muscular and from tendinous substance, and presenting distinct physical marks of a specific character. The aridity of arterial fibre, compared with the moisture of muscular fibre, is very striking. The fragility of the arterial fibre is well illustrated by the experiment of Desault. Take a portion of the aorta, and a piece of intestine, and surround each with a ligature. On tightening the ligature which surrounds the artery, we find that its middle and internal coats are cut through, as if they had been divided by a knife, while the external coat remains entire. The ligature round the piece of intestine merely brings its sides together, but never divides any of its tunics, however tightly it may be drawn.

2. When exposed, along with the other organs, to air and moisture, the arteries are found to putrify with much more difficulty than muscular substance. After the muscles of a limb are dissolved to a putrid pulp, the arteries may still be traced, firm and almost unaltered. Even during life this resisting texture of the arteries is rendered evident; for often, in the midst of a phagedenic or gangrenous sore, the result of inflammation or the consequence of gunshot wound, the large arteries are dissected by the disorganisation, and exposed to our view entire and uninjured.

3. Muscular fibres are in every animal essentially white. The blood which sojourns in the human muscles of locomotion, may be entirely removed by washing with water, and then they present the very same appearance as the muscular fibres of the hollow viscera. But the fibrous tunic of the arteries is of a yellowish color, and cannot, by any degree of washing, be made to assume the color of the muscular fibre.

Now these and some other particulars which might be mentioned, even taken collectively, may not be sufficient to constitute an absolute physical test of the two substances in question. It is possible to conceive muscular fibres of a yellowish color, dry and brittle in texture, and very resistant of putrefaction; but fibres such as these, plainly exercising muscular powers, have never as yet been discovered in any part of any animal. Knowing particularly that all muscular fibres are soft and lax, and supplied with a large quantity of fluid, we can scarcely be persuaded to admit the possibility of fibres so destitute of these properties, possessing the power of muscular contraction. It is sufficient to compare a section of the œsophagus with a section of the aorta, to be struck with the difference: the sides of the one falling together, and its substance sliding under the finger in every direction; the other stiffly gaping, from the elasticity of its texture, and appearing more like a cylinder of cartilage than a muscular tube.

II. The second argument against the muscularity of arteries, which this writer professes to examine, is "that no kind of stimuli can make arteries evince a contractile power;" or, in other words, that arteries are destitute of muscular irritability.

Under this head, we might expect that the writer would bring forward proofs of the fact; that he would show that, either in his own experiments, or in those of others, the middle coat of the arteries was agitated by muscular contraction, as all acknowledged muscles are in every part of every animal, on being submitted, during life or soon after death, to mechanical and chemical irritants, or to the electric or galvanic influence. Aware that such experiments have been tried in vain,—that we can dissect the artery of a living animal coat after coat, or expose the arteries to the electric or galvanic influence, without exciting the slightest convulsion—the most obscure muscular palpitation,—he proceeds to another method of combating this unanswered and unanswerable argument, that of confounding contractility of tissue and muscular irritability; and roundly tells us that "Bichat maintains that arteries possess no contractile power!"

Let us hear what Bichat himself says. "The contractility of the arteries," says that illustrious author, "is much more marked than their extensibility. As soon as an artery ceases to be distended with blood, it manifestly contracts. To this contracting of the arteries, the following phenomena are to be attributed:—1. The umbilical arteries and the ductus arteriosus turn, after birth, into ligaments. 2. If we tie an artery, all that part of the tube contained between the ligature and the first collateral branch is obliterated, as is proved by the operation for aneurism. 3. If we inclose a portion of the carotid between two ligatures, and then empty it by a puncture, it immediately loses half its diameter. 4. In the dogs into which I transfused blood, in order to produce an artificial plethora, I observed the arteries to have a diameter almost double that which the arteries presented in dogs of the same size, from whom I had withdrawn a large quantity of blood. 5. Experiments have perfectly satisfied me respecting the cause of the fulness and smallness of the pulse. The artery is unquestionably larger or smaller, according to the quantity of blood with which it is filled. There is a point of extension which arteries never exceed; but they often contract, from scantiness of blood, till they are all but threads. 6. In the dead subject, we are astonished that, in individuals of the same size, the arteries present such different diameters. That depends entirely on their state at death: if, from deficiency of blood, the arteries had remained long contracted, they continue in that state. 7. In a longitudinal wound of an artery, the edges of the fibrous circles which have been divided, separate from one another, and a gap, which never unites, remains between them."*

Such are the facts brought forward by Bichat, to prove the contractility of the arteries,—by Bichat, whom this gentlemen represents as maintaining that "the arteries possess *no contractile power!*"

This writer notices the effects of cold, either directly applied in the form of air to an exposed artery, or indirectly by cold bathing of the skin. Both these applications constringe the arteries, whence he concludes that the arteries must be muscular; not reflecting that the same argument will prove the muscularity of the skin, and indeed of every living fibre. Every structure possessing contractility of tissue is constringed on exposure to cold.

As for the experiment of Dr. Hales, it by no means proves the muscular irritability of the arteries: it only illustrates their

* Bichat *Anat. Gen.* partie i. tome ii. p. 307.

contractility of tissue. Brandy, there can be little doubt, will constringe arteries more than cold water, and cold water more than warm. This effect will take place even in the dead animal, and will not be confined to the coats of the arteries, but extend even to the nerves, to the cellular substance, and to every fibre of the body.

From such experiments it is rightly concluded that the arteries are possessed of a contractile power; but, to account for this power, we have no need to suppose the arterial fibres to be muscular. His reasoning amounts to this:—Every part, possessing a contractile power, is muscular; the arteries possess a contractile power; therefore, the arteries are muscular. We might as well say, the veins and the skin are possessed of a contractile power, therefore they are muscular. Throughout the whole of his paper, this is the invariable line of argument: The arteries are contractile, can inert tubes contract? The arteries are possessed of a contractile power, therefore they are muscular.

He notices under this head the peculiar mobility of the iris, a part, of which the asserted muscularity is still a matter of discussion among physiologists, and appears to be attended with more than one insuperable objection. The extensive motions of the iris serve to separate it widely from muscular structure.* Indeed, there is no example of so great a degree of extension and contractility in any acknowledged muscular part of any animal.

III. This writer next turns to the fact, that the middle coat of the aorta is not a continuation of the left ventricle of the heart. Now this, as far as I know, has never been advanced as an *argument* against the asserted muscularity of the arteries.

The facts are, the lining membrane of the heart (or sanguiferous membrane, as I have been in the habit of calling it, and which is continued, in one form or another, into every part of the body,) enters the aorta, forms by its duplication the semilunar valves, and proceeds to line the arterial system. This membrane is the sole *immediate* mode of union between the artery and the heart. The fibrous coat of the aorta does not identify itself with the muscular fibres of the heart; there is even an interval of two or three lines between the edge of the ventricular outlet and the beginning of the fibrous coat of the artery. This entire insulation of the aortic fibres from those of the heart, was particularly noticed by Bichat, and stated as the ground of a strong *presumption*, merely, that the nature of the two parts was different.

The auricles and ventricles resemble each other so strictly in their physical properties, that, if we present them to a common

* We believe that we have given this sentence correctly: unfortunately, the MS. was nearly obliterated.—Editors.

observer, he at once pronounces them to be flesh; if we submit them to an anatomist, he pronounces them to be formed of muscular substance. Besides, they are not merely similar, they are closely bound together. But expose the root of the aorta with the neighbouring part of the left ventricle, the common observer remarks at once the striking difference of their physical properties, and cannot readily be brought to admit that they are the same substance. Their lining membrane, indeed, is similar, and it is continued from the one to the other. Why should not the middle coat of the artery be continued also from the ventricle, if they are of the same substance? This abrupt commencement of the middle coat, and its very different appearance from the flesh of the heart, are presumptions, even to the common observer, against the muscularity of the aorta, and still more so to the physiologist, who will therefore examine with rigor the arguments in favor of that doctrine.

IV. This writer next notices the argument drawn from the chemical analysis of the arterial substance. He represents the analysis as imperfect, and the whole proof derived from such an investigation as obscure and nugatory. It should be sufficient authority with most men, that Berzelius and Young are of an opposite way of thinking,—that they reckon the analysis perfect, and the argument strong and incontrovertible. "It is beyond all doubt," says the former of these philosophers, "that the fibrous membrane of the arteries cannot be a muscle; for, while the latter is soft and flaccid, and contains more than three-fourths of its weight of water, the artery is dry and very elastic. The muscular fibre possesses the same chemical properties as the fibrin of the blood; for instance, that of being soluble in acetic acid, and of forming scarcely soluble compounds with sulphuric, nitric, and muriatic acids: but the arterial fibre has altogether opposite qualities,—viz. that of not being soluble in acetic acid, but pretty easily soluble in mineral acids, diluted with water to a certain degree, from which solution it cannot be precipitated by means of alkali, or alkaline prussiats, which are the tests for the acid solution of fibrin, &c. Consequently, as the arterial fibre neither has the structure of a muscle, nor its chemical properties and composition, it cannot be a muscle, nor perform the functions of a muscle; which is, besides, sufficiently evident from its elasticity."*

V. The fifth particular to which the writer in the Edinburgh Journal directs our attention, is the ossification of the arteries. And here, as every where else, he betrays his anxiety, not only to make the most of every argument in his own favor, but also to slip from the grasp of whatever arguments stand up against him. As he has made so much use of Mr. Allan Burns's work

* BERZELIUS's *View of the Progress and present State of Animal Chemistry*, p. 25.

on the Heart, in drawing up his paper, why not quote the following observation by that gentleman? "In a person, whose body I dissected some time ago, I found the heart and aorta perfectly healthy; but the arteries of the head, pelvis, legs, and arms, were almost entirely ossified. In the arms, in the arterial trunks, the circles of bone are complete, and are not above the hundredth part of an inch distant from each other. In the lower extremities, the trunks are only partially affected, but the primary branches are nearly obliterated; they are not only ossified, but their inner surface is coated over with a lymphatic-looking crust. They are so much ossified, that it is perfectly impossible that they could have assisted in circulating their contents: nay, from the incrustation in their canals, the blood would even find difficulty in moving along."*

The writer, whose opinions I combat, admits that the arteries have been partially ossified, without deranging, to any great extent, the circulation of the blood; but the ossification of the arterial system extends, in some cases, much further than he is disposed to admit. There are examples of the trunk of the aorta, (the second ventricle, as some favourers of the muscularity of the arteries have called it,) being wholly ossified;† and, in one case so rigidly, both in its ascending and descending branches, as to compel the sufferer to maintain an erect position.‡ Dr. Heberden relates the case of a very old man, who at last died suddenly, in whom the mitral valves and the semilunar valves of the aorta were ossified; as were also the coronary arteries of the heart, the thoracic and abdominal aorta, with all their primary branches, the internal carotids and basilary artery, with many of their primary branches, and the external and internal iliacs; yet the patient appeared, almost to the last, to be of a sound constitution, and free from the usual infirmities of age, being upwards of eighty when he died.§

Now, in such cases as these, and even in ossifications much less extensive, it is difficult to conceive how the circulation could be continued, if the arteries acted by muscular contraction in the propulsion of the blood; and still more difficult to conceive how the pulse could continue regular and uniform; for, though changed in point of strength, neither is its frequency nor its uniformity over the system affected.

Did the arteries actively assist in the circulation, we should expect, when they became no longer a continued set of contractile tubes, but rather a chain of osseous cylinders, that the flow of blood, urged on by the portions which still continued healthy,

---

* BURNS *on Diseases of the Heart*, p. 124.

† BUCKNER, *Miscel.* 1727, p. 305.

‡ GUATTANI *de Aneurismatibus.*

§ *Med. Trans.* vol. v. p. 174.

would become vibratory, uncertain, and irregular. Nothing of this kind, however, takes place.

*Arguments for the Muscularity of Arteries.*

This writer next proceeds to enumerate some arguments in favour of a muscular action in the arteries.

I. His first assertion is, that "the pulse is not uniformly synchronous with the contraction of the left ventricle of the heart."

This assertion is not, in the slightest degree, borne out by the four cases to which he refers. Indeed, one of them turns out not to be a case at all, but a mere conjecture by Dr. Mason Good, that, in whitlow, the radial artery, may pulsate 100, while the other arteries pulsate 70; and that, if ten different inflammations existed in one person, there might be ten pulsations in different parts of the body, all differing from the pulsations of the heart.* All this, it is evident, is quite conjectural; or, rather, it is an error, arising from too hastily considering the throb or palpitation in whitlow as a more frequent pulsation of the arteries.

The phenomenon of the double pulse attendant on contraction of the left auriculo-ventricular opening, has been perfectly explained by Mr. Hodgson. "The auricle," says he, "first acts, and propels its contents towards the ventricle; but, from the constriction of the communication, the blood is not, as in the natural state, poured at once into that cavity. The ventricle, though imperfectly filled, contracts, and forces the blood into the aorta. Thus there is a pulse caused by the action of the auricle, and another by that of the ventricle; so that, for every pulsation at the wrist, two are perceptible at the heart. Nor is the auricular pulsation trifling; for, in this disease, the collection of blood in the auricle causes it to dilate, and its parietes become thick and very muscular."†

I do not find, in Mr. Burns's work on the Heart, any case of ossification of the aortic valves producing double pulsation of the heart. He merely mentions, among the symptoms in general, that the pulse is intermitting and variable, discordant, and not equally frequent with the contraction of the heart, when the person is fatigued. The case by Dr. Balmanno, given by Mr. Burns, merely corroborates the views of Mr. Hodgson. But, even granting that such was the fact, how could it, in the smallest degree, go to prove the muscularity of the arteries? This writer admits that the pulse at the wrist is partly the effect of the contraction of the left ventricle.‡ But, in these cases of

* *Study of Medicine*, vol. ii. p. 16.
† *Treatises on the Diseases of Arteries and Veins*, p. 33.
‡ Note, p. 263.

double pulsation, nothing more is felt at the wrist than in ordinary health,—a pulse synchronous with the contraction of the ventricle; the double pulse is felt only at the heart. Were it the reverse of this,—did the heart beat one, and the wrist beat two,—there might be some reason for stating this as an argument for an arterial pulse, independent of the heart.

Lastly, under this head he quotes the cases of Dr. Parry, in which the pulse at the wrist was so feeble that it could not be felt, while the pulse in the carotids was full and strong. Various circumstances may cause the number of sensible pulsations in arteries to fall short of that of the sensible strokes of the heart. "Were we, however," says Dr. Parry, "unable to point out all these causes, that would be a curious kind of argument by which we should infer that, because a pulse was sometimes wanting in an artery, therefore the pulse, when existing, was produced by the artery. Just as legitimately might we conclude that the tube of a syringe propelled the fluid within it, because, though the piston continued to act, the fluid would no longer pass. In both cases, a certain condition of the conduit may be necessary, although in both the conduit is equally passive."* All the cases in which the remote arteries of the extremities cease to pulsate, while the pulse of the carotids continue, may be referred to one or other of these three causes,—1, weakness and disorder of the heart; 2, scantiness of blood in the system; 3, obstruction in one or more of the arteries. In regard to the two former of these causes, it may be remarked, that the carotids uniformly lose their pulse last, and, on the return of suspended life, recover it first,—both in cases of general debility from internal diseases, and in cases of hemorrhagy. The momentum of the blood is not sufficient, under these circumstances, to produce the sensation of a pulse in the radial artery, and still less in the arteries of the lower extremities: we feel, therefore, for the brachial artery or for the carotid, or apply our hand over the heart itself. In these cases, too, we cannot doubt that the arteries accommodate their diameter to the stream with which they are supplied, and contract so as to continue in contact with their contents. As for local obstruction of an artery, it may depend on inflammation of its tunics, deposition of fibrin within its cavity, ossification, the separation of ossific concretions, which not unfrequently are found projecting into the tube of arteries, and sometimes lying loose and separated; the pressure of neighbouring parts in a state of disease or displacement,—such as tumors, dislocations, and the like.

* *Experimental Inquiry into the Nature, Cause, and Varieties, of the Arterial Pulse.*

II. This writer's second assertion is, that "the arteries, after death, are always found empty."

The inference which we should naturally draw from this assertion is, that the emptiness is owing to the muscular action of the arteries themselves. We find, however, as we proceed through the argument, that the arteries are not always found empty after death; and that these vessels, however strong their muscular power, are of themselves unable to produce the emptiness when it does occur.

A different situation is assigned to the mass of circulating blood, according as death primarily affects the brain, the heart, or the lungs. After sudden death, whether it arises from disorder of the brain or of the heart, the arterial system is always found more or less filled with blood: but, if the process of death has been slow, the lungs and the venous system become the depositaries of the blood, and the arteries are found empty. The longer the agonies of death have lasted, the lungs are found the more voluminous and the heavier, from the quantity of blood with which the branches of the pulmonary artery are gorged. By far the greater number of deaths take place in this way. Whatever may have been the original seat of the disease,—whether it has been some local affection or some general disorder,—it is always in the lungs that the terminating embarrassment begins: the respiration becomes difficult, the arterialization of the blood gradually ceases to be effected, sensation, intellect, and voluntary motion cease: but the heart still continues to beat,—it is truly, in this kind of death, the *ultimum moriens.* The left side of the heart receives less and less blood from the lungs, and consequently sends less and less into the arteries. The anterior and posterior sides of the descending aorta, and of its larger branches, fall together from emptiness; and in this state they are found on dissection. But the pulmonary artery, the right cavities of the heart, and the systemi veins, are filled with blood; so that, on dissection, they are found more capacious than we have any reason to suppose they ever are during life.

On the other hand, when the heart is struck with sudden disease, so that its action is the first arrested in the process of death, the functions of the brain and the whole of the voluntary functions rapidly cease, and speedily the function of the lungs also. On opening the dead body, the lungs are found of their natural colour, collapsed, and not at all gorged with blood. This fluid is found, after this kind of death, distributed through the body,—the arteries being filled with arterial, and the veins with venous blood. This is found to be the state of the parts in all cases of disease terminating by fatal syncope; an event very frequent in cases of dropsy, hemorrhage, and various other diseases.

When the brain is the first seat of the process of death, the following is the train of phenomena :—Sensation and voluntary motion suddenly cease; the diaphragm and intercostal muscles are struck with palsy; the mechanical part of respiration ceases, and speedily the chemical part also. The heart stops early or later, according to the rapidity with which the process of respiration has been interrupted. After death, we find a quantity of venous blood in the aorta and large arteries; the venous system more or less filled, as the process of death was slow or rapid; the lungs empty, if the death was sudden,—gorged, if that process had been prolonged.

How do these facts affect the question of the muscularity of the arteries? All that can be proved from them appears to be this, that, when the heart suddenly ceases to contract, the blood is arrested in the blood-vessels, and cannot be propelled into the veins by any action of the arteries themselves; a fact which goes against the opinion that these tubes are muscular, and actively propel the blood.

On the contrary, when the heart remains active to the last moment, no blood is found in the arteries; it is accumulated in the venous part of the circulating organs. Even the last gush of blood coming from the left ventricle, tarries not in the arteries, but passes on into the veins.

The active agents of this transition appear to be the contracting left ventricle on the one hand, and the dilating right auricle on the other. That the contraction of the left ventricle is extremely powerful, is demonstrated by many striking facts. When, in the living body, we open an artery no bigger than a pin, the blood springs from it to the distance of three or four feet, with such force, that Mr. Charles Bell has thought it necessary, in order to explain so powerful an effect from the impulse of the heart, to suppose that, between the blood and the sanguiferous membrane, there was an absence of that attraction which is universal in dead matter. From the momentum which the blood receives from the left ventricle, arises the pulse in every part of the body. On the other hand, the right auricle dilates, not so much by the impelling power of the blood of the venæ cavæ, as Harvey appears to have supposed, as by a spontaneous elongation of its muscular fibres; for, in experiments on living animals,—for example, in those of Mr. Brodie,—this dilatation of the auricle goes on alternating with its contraction, for some considerable time after the heart is entirely empty of blood.* The mere dilatation of the right auricle, by producing

---

* Cooke *on Nervous Diseases*, vol. i. p. 61.

a tendency to a vacuum, acts in pumping the blood from the venæ cavæ.

The momentum, then, communicated to the unda of the blood by the left ventricle, assisted by the contractility of tissue of the arteries, transmits the blood into the venous system, prevents the veins from collapsing, and regularly places a fresh supply of blood within the sphere of the soliciting influence of the right auricle.

Dr. Carson has shown that, during life, the lungs must act in a considerable degree, from their resilience or contractility of tissue, as antagonists of the heart. The lungs have a constant tendency to return to the state in which they were before respiration began: this resilience, elasticity, tendency to collapse, or contractility of tissue, is opposed by the weight of the atmospheric air flowing down the windpipe; and, on the other hand, this resilience favours the dilating, and opposes the contractile power, of the heart. He supposes that the resilience of the lungs possesses an uncontrolled operation after death, and that the consequence is a tendency to the formation of a vacuum in the chest. But, to fill up this vacuum, there is a draining of the blood from all parts of the body; and hence a large quantity of blood is collected in the neighbourhood of the heart after death. He asserts that the heart and large vascular trunks, being all within what may be called the vacuum of the chest, are distended to their utmost capacity; and that the additional blood requisite for this purpose is drawn, through the veins, from those parts of the vascular systems not situated in the chest.*

These theoretic views of Dr. Carson, respecting the causes of the vacuity of the arteries after death, are no doubt ingenious; but they appear insufficient to explain the different effects of the different kinds of death.

The writer in the Edinburgh Journal, however, has entirely misunderstood Dr. Carson's views. He has supposed resilience to mean dilatation, and not contraction, with which, in Dr. Carson's writings, it is synonymous. Labouring under this misapprehension, he relates four experiments of his own,—the last of which consisted in tying the aorta of a cat, at the same time, through the windpipe, inflating the lungs, in order to give their resilience every fair chance! "Upon considering the matter," says he, speaking of his first three experiments, "I remembered that the lungs, from the entrance of the atmospheric air into the chest, had been, during the whole experiment, in a state of collapse. In the fourth experiment, this objection was

* Carson's *Inquiry into the Causes of the Motion of the Blood,* p. 108, 131, &c. —*Med.-Chir. Trans.* vol. xi. p. 165.

obviated by inflating the lungs; and the result was such as has been mentioned,"—the arteries were found perfectly empty. "The vacuity of the arteries after death, then, is attributable to three causes:—1st, to the contraction of the arteries; 2dly, to the suction-power of the right side of the heart; and 3dly, to the natural resilience of the lungs, producing a tendency to a vacuum."

Such is this writer's mistake regarding the doctrines of Dr. Carson. Dr. Carson thinks that the resilience, or collapse, of the lungs favours the formation of a vacuum in the chest, which in its turn favours the attraction of the blood from all parts of the body, into the heart and large vessels, after death. This writer supposes resilience to mean dilatation, and finds his experiments perfectly to succeed, when he lustily inflates the lungs, and confines the air by putting a ligature round the windpipe!

III. This writer next attempts to show, that occasionally the heart is so changed by disease, that it is incapable of assisting in the circulation, which must therefore be carried on, in such cases, by the arteries alone. There happens, however, to be no case on record, in which both the left auricle and ventricle were so changed by disease, that they could not carry on the systemic circulation.

Mr. Burns's case of William Brown is very imperfectly quoted. This writer states that the affection of the heart in this man was frequently so urgent, that he dropped down apparently dead. Mr. Burns says merely that, twice during intoxication, the affection of the heart became so urgent, that he dropped down. This writer states that, on inspecting the body, the ventricles of the heart were found completely changed in structure, being now composed of a substance intermediate between fat and cartilage; but he takes no notice of the important fact mentioned by Mr. Burns, that the auricles had their musculi pectinati much enlarged, and redder than usual. The omission of this fact makes an essential difference, and, if intentional, was uncandid. The auricles were not merely free from disease in this case, but were actually more muscular than usual; thus making up for the deficient power of the ventricles.

This writer asserts that several cases of the heart converted into solid bone, will be found by referring to Burnet, Baillie, Good, Bordenave, Simmons, and Burns: I assert, on the contrary, that not even one such case is related by these authors; nor, as far as I know, by any other author.

Good relates no case of ossified heart which had occurred to himself, but merely quotes from Burnet, Baillie, and Morgagni.

Under the head of "Heart bony, or earthy," in his Morbid Anatomy, Dr. Baillie says, "Neither of these appearances has come under my own observation, and they are to be looked upon as very uncommon." He refers us to Bonetus and Morgagni.

BURNET mentions several cases of calculous concretions in the cavities of the heart; but nothing more.*

BONETUS relates several cases of bits of calcareous substance being found in the heart's cavities, and in the substance of its parietes; but nothing more.†

MORGAGNI found a bit of bone near the mitral valve in one heart, and two osseous scales adhering to the external surface of another.‡

In BORDEMANE's case, the pericardium was thickened, and firmly united with the heart, *on the surface* of which was a bony concretion of unequal thickness, and in some places two inches in breadth. This ossification covered almost the whole of the right ventricle, from the basis to the apex of the heart, and, following the course of the septum, ascended from the apex about half way up the left ventricle, leaving the auricles untouched.

"In the case related by Dr. SIMMONS," says the writer in the Journal, "the ossification extended from the base to the apex of the heart, and consequently involved *both auricles and ventricles.*" I cannot bring myself to believe that this writer consulted the case, as it is related by Simmons and Watson, in the first volume of the Medical Communications. But how could he make such an assertion as the above, even if he had taken the case at second-hand from Mr. Allan Burns, when this author expressly adds to his very abridged account of it?—"From the change of structure being partial, and also from its position, it would not materially impede the action of the auricles. This I infer from the sinus venosus and auricular portion of the auricle being unaffected."

When we turn to the case as related by Simmons and Watson, we find that the ossification was situated on the flat surface of the heart, the centre of it nearly corresponding with the septum of the ventricles; that it was broadest in the middle, and branched irregularly through the fleshy substance of both ventricles, over the greater part of the right auricle, and for some way round the basis of the ventricles, following the course of the coronary vessels. It no where projected into the cavities of the heart; and appears, from the figure given of the heart, to have been superficial, and even less extensive than we should be led, from the description, to suppose.

Dr. Baillie, in his Series of Engravings, has re-engraved this

* THESAURUS *Medicinæ Practicæ*, p. 230.

† *Sepulchretum*, vol. i. p. 820 and 825.

‡ *De Causis et Sedibus*, Ep. iii. § 2, xxvii. § 16.

heart;* and, in his explanation of the plate, mentions one or two particulars, which are interesting. The descending aorta, he observes, was smaller than the natural size. "This," says he, "had been occasioned by the blood being propelled into it from the left ventricle, in less than the natural quantity, and by a feeble exertion." Had the heart been altogether useless, as the writer in the Journal would have us to believe, and had the blood been circulated by contractions of the aorta, it is probable that the artery would have been increased both in thickness and diameter. Dr. Baillie adds, "The heart from which this figure was taken appeared somewhat larger than the natural size; and this may have arisen from the blood being accumulated in it, in consequence of the extent of the ossification, which was so great as to prevent the *full degree* of contraction." He describes the ossification in these words:—"A large, irregular ossification, covering a considerable part of the right ventricle and of the right auricle, rendering the limit between the two in some degree indistinct." These several remarks are all confirmatory of the fact, that the blood was driven from the left side of this heart, by an auricle entirely free from ossification, and by a ventricle ossified on part of its external surface only.

The case of partial ossification of the heart of Margaret Henderson, related by Mr. ALLAN BURNS, this writer treats as he does the last. He contents himself with mentioning that "the whole extent of the pericardium which covered the ventricles, and the ventricles themselves, except about a cubic inch at the apex of the heart, were ossified and firm as the skull;" and omitting altogether to state that "*both auricles were healthy, but thicker than usual.*" Mr. Burns mentions, too, that this patient's pulse, ten months before her death, was of unequal strength, and, for some days before death, feeble and intermittent; which this writer omits to notice.

Having thus given but a partial account of the cases in question, this writer proceeds to draw the conclusion that, in such cases, the heart must have been useless for years: "yet," adds he, "the circulation was not only maintained, but maintained with such regularity, that the disease was frequently overlooked or mistaken."

Now, first, with regard to the regularity with which the circulation was maintained in the case in question. Mr. Burns's first-mentioned patient "had for many years an affection of the heart." In Bordemane's case, the symptoms were considerable difficulty in breathing, a peculiar anxiety and feeling of suffocation, and a slow and feeble pulse. The consequence of the disease in Simmons's case, are stated to have been irregular and

* Fasciculus i. plate 5.

feeble action of the heart; the blood poor and watery; the few coagula found on dissection very tender; all the solids bearing evident marks of a lax fibre; the muscles pale and flabby; the viscera soft; the hollow ones thin, weak, and almost transparent; the fat soft and putrescent: in a word, all the effects that might be supposed to arise from a disordered and languid circulation. In Mr. Burns's second case, the symptoms were cough, much difficulty in breathing, œdema of the extremities, dropsy of the belly, enlargement and induration of the liver, severe attacks of dyspnœa, a livid color of the skin during these attacks, fits of nausea, great anxiety in the chest, pain diffused over the abdomen; she was compelled to continue constantly in a semi-erect position, a recumbent posture never failing to induce a paroxysm of breathlessness; the pulse of unequal strength, and at last feeble and intermitting. These are the signs from which this writer concludes, that "the circulation was not only maintained, but maintained with regularity."

In the second place, with regard to the power by which the remaining feeble and irregular systemic circulation in these cases was kept up, it is pretty evident, from what has already been said, that that power resided in the left auricle, which was healthy and unossified in every one of the cases alluded to, and in two of them unusually thick and fleshy. "The cases of Henderson and Brown," says Mr. Allan Burns, "prove, most incontrovertibly, that the circulation may be carried on without any assistance from the ventricles; but they, at the same time, show that, in consequence of the deviation from the natural function, the vascular actions are precarious and irregular. The auricles are of themselves competent to the performance of the offices of the whole heart; but then they can only accomplish this while the circulation remains undisturbed. In the history of these cases, you have noticed that every increase in the celerity of the circulation was followed by an impaired state of the whole system. Of this, the cause is obvious: the auricles were excited to a degree of action incompatible with their power; and this, in both cases, was urged so far as to terminate in a total cessation of action."*

IV. The writer in the Edinburgh Journal next directs our attention to the comparative anatomy of the apparatus of circulation.

He speaks particularly of the circulation in insects, probably

---

* Burns *on Diseases of the Heart*, p. 135.—M. Soumain's case is not referred to by the writer in the Edinburgh Journal. I have said nothing of it, because I have not had an opportunity of consulting the original account of it. In that case, only one of the ventricles is said to have been left, the rest of the heart having been destroyed by disease. (*Histoire de l'Academie des Sciences*, 1768; tome 142, p. 102.)

not being aware that, in insects, neither heart, arteries, nor veins, have been discovered. Perhaps, having heard that they have no heart, he has concluded that their arteries must be muscular, and, of course, that the human arteries must be muscular too! Such is a too-common method of reasoning without premises. In the insecta of modern zoologists, neither absorbing nor circulating vessels have been detected. The nutritious part of their food appears to be absorbed by the walls of the intestines, and discharged into the cavity of the body, where there are neither veins, arteries, nor heart. Towards the back, indeed, there is the dorsal vessel; but which has been ascertained neither to receive motion from, nor give motion to, any circulating fluid: it is neither a heart nor an aorta.*

The only living creatures which have no central organ of the circulation in the form of a heart, and yet evidently possess the function of circulation, are some species of the *annelides*. But, though in these animals the impelling organ of the circulation has changed its form, we must by no means suppose it to be wanting. The aorta of these animals dilates, at different points of its course, into cavities, which are observed regularly to contract and urge on the blood, and which have, therefore, received from some authors the name of hearts. It is plain, from this statement, that the aorta of a leech or silk-worm bears but a remote analogy indeed to the aorta of a man, or of the other mammalia. Both in form and in action it is widely different, and is rather a chain of ventricles, than a mere conveying artery.†

Fishes have a pulmonic heart only. Their blood, after being renovated in the gills, is re-absorbed by a multitude of minute vessels, which unite into an aorta. This descends along the inferior side of the spine, in a canal fitted for its reception, and to which its external surface is firmly adherent. That the aorta of fishes does not act as the aortal heart of the annelides, may be concluded, first, from this adherence, which will necessarily prevent any considerable contraction of the vessel; and, secondly, from the aorta in fishes being uniformly full of blood in the dead animal. How then, it may be asked, is the systemic circulation carried on in fishes? The greatest of comparative anatomists has long ago answered this question:—it is by the impulse of the pulmonic heart. "For instance, the sturgeon," says Cuvier, "gives us an evident proof of the continuation of the action of the pulmonic heart on the aortic system. Scarcely have the pulmonic veins united to form the aorta, when this vessel enters the cartilaginous canal formed for it by the bodies of the vertebræ: there it is stript of its tunics, and the blood

* CUVIER's *Anat. Comp.* tome iv. p. 163. † *Ib.* p. 412.

flows in a tube, the walls of which are absolutely immoveable. By the holes of this cartilaginous tube or canal, the arterial branches come out, which go to the different parts. It is evident that the blood cannot enter these branches, but in virtue of the impulse which it has primitively received from the heart and pulmonary arteries."*

As there exists a systemic ventricle in the arachnoida, in the crustacea, in all the mollusca, in all reptiles, in birds, and in the mammalia, it is evident that the only ground for an argument on this question, derived from comparative anatomy, is to be found in the structure of fishes, and worms with red blood. But the objection derived from these animals is completely removed, when the structure and action of the different parts of their circulatory apparatus is examined. The annelides have not one systemic ventricle, but many; while the ventricle of fishes is at once pulmonic and systemic.

Under this head, the writer in the Journal so frequently alluded to says, that "monsters have been born without a heart, and have lived for some time, the blood circulating with apparent regularity." I have met with no such case on record. Mr. Lawrence, in the view which he has given of the principal monstrosities, and of the physiological inferences to which they lead, has not mentioned any such case.† Under the head of *Monstrosum*, Ploucquet notices no such case. Cases, no doubt, are on record of fœtuses wanting a heart, or wanting even the upper half of the body; but no instance, I believe, will be found recorded, in which fœtuses, so imperfectly formed, gave any sign of independent vitality. On the contrary, they evidently belong to Mr. Lawrence's second class, and are totally incapable of supporting life after birth.

V. The last topic on which this writer touches is, that the vessels of the skin become, in some cases, as it were instantaneously filled with blood,—for instance, in shame; and in other cases are almost as quickly emptied,—for instance, in fear.

That the heart in both these cases is affected, I need scarcely attempt to prove. How vivid soever the sensations which give rise to the appearances in question, it cannot be doubted that they affect the skin only through the medium of the nervous system operating on the heart, and ultimately on the capillaries. The chain of intermediate causes is, indeed, not an inconsiderable one, but it is struck by the electric touch of life.

Why this sudden filling and emptying of the cutaneous vessels is seen most upon the face, is explained by the well-known anatomical fact, that the capillaries of that part are more

---

* Cuvier *Anat. Comp.* tome iv. p. 177.

† *Med.-Chir. Trans.* vol. v. p. 165.

dilatable than those of any other parts of the integuments, and more under the influence even of any dead force filling the aorta with fluid. Fine injection colors the face more than the other parts.

The arteries, according to Bichat, are the vessels through which the blood passes by the influence of the heart. They are strangers to the structure of the organs of the body; they wind into the interstices merely of these organs, and are every where imbedded in the cellular membrane: but the capillaries form an essential part of many organs, and they enter into the ultimate composition of every structure; and every atom of nutriment or of secretion is deposited by them.

The structure of the capillaries is unknown, and may be very different from the structure of the arteries. The capillaries are too minute to allow of their being separated from the parts with which they are interwoven, so that it is impossible to institute any analysis of their substance, or even to demonstrate their physical properties. That the circulation through the capillaries is independent of the circulating power of the heart; that the blood, once arrived in the capillaries, is beyond the influence of this organ, and circulates by a power or powers to us insensible and unknown,—is a doctrine to which I cannot altogether subscribe; and yet it is supported by some facts, which I should think is uncandid to overlook. The motion of the fluids in the capillaries does not, in all cases, correspond with that of the blood in the arteries; the augmentation of nutrition and secretion does not correspond with the augmentation of the heart's action; nor does nutrition or secretion diminish in proportion to the diminution of its action. On the contrary, in the most violent fits of fever, when the heart and arterial circulation are in a state of the greatest agitation, the capillaries are often inactive, nutrition flags, and the secretions are ch[illegible]ked. As the febrile paroxysm diminishes, the capillaries begin to re-act, and the natural exhalations and secretions return. How often do we see hemorrhage from the capillaries, when the heart is in a state of extreme weakness? How often do we see the exhaling capillaries pouring out large quantities of fluid into the cavities or upon the surface of the body, while the heart is scarcely able to follow up one feeble labouring pulsation by another? There is little or no harmony, in such cases, between the capillaries and the heart. They have then, in a certain degree, and under certain circumstances, an independant action.

But all these considerations, which have been set forth with so much eloquence by Bichat, can never prove the aorta and the arteries to be muscular. Against this doctrine, we have already seen that there are hosts of arguments, which no known facts can overturn.

*Three additional Arguments against the Muscularity of Arteries.*

I. The arteries exposed in a living animal betray no signs of contraction or relaxation.

If the aorta be as it were "a second heart,"* urging on the blood by muscular contractions,—if all the arteries are muscular tubes, assisting in actively propelling the blood,—the vessels will necessarily present a systole and diastole, similar to the contraction and relaxation of the auricles and ventricles of the heart. But, if we expose the aorta, or any of the large arteries, such as the carotid, in a living animal, though the blood continues to go from the heart and return to it, as in the natural state of the parts, no contraction or dilatation of the exposed arteries is dicernible. On taking the exposed artery between the finger and thumb, the momentum which the blood receives from the left ventricle is felt to produce a strong pulsation; but there is not the least sign of any action in the arteries themselves, urging on the tide of blood.

This argument has been fully established by the experiments of Dr. Parry.

II. The walls of the left ventricle are more than twice thicker than those of the right ventricle.

Were the aorta and its branches muscular, and sufficient of themselves to carry on the circulation into the capillaries, it is not probable that the two ventricles should be so strikingly disproportionate in point of muscular strength. Indeed, upon this supposition, this surprising fleshiness of the left ventricle would be but a waste of the material. But if the right ventricle, by its contraction, has to force the venous blood merely through the lungs, while the left, by its contraction, has to force the pure blood into the remotest parts of the body, the striking inequality is perfectly explained.

III. The asserted muscular contractions of the aorta are not alternate, and they cannot be synchronous, with the contractions of the left ventricle.

This is an argument which I have always stated in my Anatomical Lectures, in the following manner:—The contraction of the auricles is not simultaneous with that of the ventricles, but successive or alternate. Had both auricles and ventricles contracted at the same instant, it is plain that the auricles never could have emptied themselves into the ventricles. Those who maintain the muscularity of the aorta and its branches, appear to me to hold an opinion equally false as if they had maintained that the auricles and ventricles contracted together. No one doubts that the pulse is the effect of the contraction of the left

* Bell's *Anatomy*, vol. ii. p. 88.

ventricle, and that it is synchronous with the contraction of the ventricle. Now, if the aorta be muscular, and contract like another ventricle, its contraction must either be alternate with that of the ventricle, or simultaneous. If it contracted alternately with the left ventricle, the pulse would be doubled in number,—the blood would betray not merely the momentum it receives from the ventricles, but also that which it would receive from the aorta. If the aorta contracted simultaneously with the ventricle, the latter could never empty itself of blood into the aorta.

