# Errata in No. 312

**Published:** 1825-04

**Authors:** 


					Errata in No. 3l2.~
Page 1)5, for Balmanus read Balmano. P. 99, for uncon-
-  -S o" Wttuuuuu. ?tH?? uuiiuaiivi MT.yyjJVTUlIUUII-
Iron lea read uncontrolled. P. 104, for converging read conveying. P. 106, for
choked read checked. P. 100, for Bordeman eread Bordenave.

				

